# MdMYB6 regulates anthocyanin formation in apple both through direct inhibition of the biosynthesis pathway and through substrate removal

**DOI:** 10.1038/s41438-020-0294-4

**Published:** 2020-05-02

**Authors:** Haifeng Xu, Qi Zou, Guanxian Yang, Shenghui Jiang, Hongcheng Fang, Yicheng Wang, Jing Zhang, Zongying Zhang, Nan Wang, Xuesen Chen

**Affiliations:** 1 College of Horticulture Science and Engineering, Shandong Agricultural University, 61 Daizong Road, Tai’an, 271018 China; 2 State Key Laboratory of Crop Biology, Shandong Agricultural University, 61 Daizong Road, Tai’an, 271018 China

**Keywords:** RNAi, Plant molecular biology, PCR-based techniques, Transcriptional regulatory elements

## Abstract

Anthocyanin biosynthesis and sugar metabolism are important processes during plant growth, but the molecular interactions underlying these pathways are still unclear. In this work, we analyzed the anthocyanin and soluble sugar contents, as well as the transcript levels of transcription factors that are known to be related to the biosynthesis of anthocyanin in ‘Hongcui 1’ apple flesh during fruit development. Overexpression of *MdMYB6* in red-fleshed calli was found to reduce anthocyanin content and result in downregulated expression of the MdANS and MdGSTF12 proteins. Yeast one-hybrid and electrophoretic mobility shift analyses showed that MdMYB6 could directly bind to the promoters of *MdANS* and *MdGSTF12*, indicating that MdMYB6 could inhibit anthocyanin biosynthesis by regulating *MdANS* and *MdGSTF12*. Overexpression of *MdTMT1* in the *Arabidopsis tmt1* mutant restored the glucose and fructose contents to the wild-type levels, while overexpression of *MdTMT1* in red-fleshed calli increased the contents of glucose and fructose but reduced the contents of UDP-glucose, UDP-galactose, and anthocyanin. Using a GUS reporter system, yeast one-hybrid, chromatin immunoprecipitation-PCR and electrophoretic mobility shift analyses, we found that MdMYB6 could bind to the promoter of *MdTMT1*, resulting in increased promoter activity. Overexpression of *MdMYB6* in calli overexpressing *MdTMT1* increased the expression of *MdTMT1*, which led to reduced contents of UDP-glucose and UDP-galactose and decreased anthocyanin content compared to those of the calli that overexpressed *MdTMT1*. This finding suggested that MdMYB6 could also inhibit anthocyanin biosynthesis by regulating *MdTMT1* to decrease the contents of UDP-glucose and UDP-galactose. Taken together, these results showed that MdMYB6 and MdTMT1 play key roles in both anthocyanin biosynthesis and sugar transport.

## Introduction

Anthocyanins are important secondary metabolites in plants and are mainly distributed in the vacuoles of plant tissues, including seeds, fruits, flowers, leaves and other organs^1^. Anthocyanins accumulate at high levels in some mature fruits, such as grapes, strawberries, apples, red pears, mangosteen and blood oranges. These pigments not only give fruits their rich colors but have also been shown to prevent cardiovascular disease, reduce obesity and improve glucose homeostasis in humans^2,3^. Anthocyanin biosynthesis involves various transcription factors (TFs) and structural enzymes, which are encoded by genes such as anthocyanin synthase (*ANS*) and UDP-glucose flavonoid 3-glu-cosyltransferase (*UFGT*)^4,5^. This process is transcriptionally regulated by the MYB-bHLH-WD40 complex, whose members have been studied extensively^6–9^. Homologs of *Arabidopsis thaliana AtMYB75* that are involved in anthocyanin biosynthesis have been found in pear (*PyMYB10*), peach (*PpMYB10*), grape (*VvMYBA1* and *VvMYBA2*), strawberry (*FaMYB10*), and apple (*MdMYB1*)^10–12^. In red-fleshed and red-foliage apple, the transcription factor encoded by *MdMYB10* can bind to its own promoter and control anthocyanin biosynthesis in apple flesh^13,14^. A novel MYB gene, *MdMYB110a*, was found to regulate anthocyanin biosynthesis in sangrado apple that has green foliage and develops red flesh in the fruit cortex late in maturity^15^. In addition, the first MYB repressor involved in anthocyanin biosynthesis is FaMYB1 in strawberry, which reduces anthocyanin content in tobacco petals when heterologously expressed in tobacco^16^. A petunia homologue, PhMYB27, was shown to act upon MBW complexes to inhibit anthocyanin biosynthesis^17^. In apple, MdMYB6 could inhibit the anthocyanin content in transgenic *Arabidopsis*^18^, and MdMYB16 could directly repress anthocyanin biosynthesis via the C-terminal EAR sequence^19^.

Anthocyanin biosynthesis is induced by plant hormones and some environmental factors, such as low temperature and ultraviolet-B radiation^20,21^. In apples, MdbHLH3 has been shown to be involved in low temperature-induced anthocyanin biosynthesis^22^, while MdCOL11 and MdBBX20 are known to control anthocyanin biosynthesis in response to ultraviolet B and low temperature^23,24^. MdMYB9 and MdMYB11 participate in jasmonic acid-induced anthocyanin biosynthesis^25^. In addition, AtTT19 and MdGSTF6 transport anthocyanins from the cytosol to the vacuole, thereby contributing to anthocyanin accumulation in plant cells^26,27^.

Sugars are the main products of photosynthesis in plants and are involved in nutrient storage and transport, signal transduction, and stress resistance^28^. In members of Rosaceae, such as apple, apricot, peach and pear, sorbitol is the main form of sugar subjected to long-distance transport via the phloem. However, fruits contain starch, sucrose, fructose, and glucose as the main sugars, and sorbitol only makes up a small portion of all the sugars. In *Arabidopsis*, there are at least 69 sugar transporters, including monosaccharide and polysaccharide transporters, belonging to eight subfamilies^29^. Sucrose transporters comprise five subfamilies, namely, SUT1–5^30,31^. SUT1 and SUT3 are mainly expressed in both source and sink tissues and are responsible for the loading and unloading of sucrose in the phloem and the absorption of sucrose by sink tissues^32^. Endler et al.^33^ showed that HvSUT2 and AtSUT4 are localized at the tonoplast and mediate the exchange of sucrose between the vacuole and the cytoplasm.

Plant monosaccharide transporters are membrane-bound proteins with 12 transmembrane domains^34^, which act to transport glucose, fructose, galactose, mannose and inositol across cell membranes and tonoplasts. In *Arabidopsis*, the monosaccharide/proton symporter AtSTP1 transports glucose and is expressed in germinated seeds, roots, and cotyledon guard cells, where it participates in sugar absorption under dark conditions in many tissues and in roots under normal physiological conditions^35^. The early response to dehydrate 6-like protein (AtERD6L) is localized to the tonoplast and transports glucose stored in the vacuole to the cytoplasm under stress conditions^36^. The tonoplast monosaccharide transporters (TMT) AtTMT1 and AtTMT2 were found to be induced by exogenous sugar treatment and are involved in vacuolar monosaccharide transport, especially during responses to salt and cold stresses^34,37^. Members of the TMT subfamily have phosphorylation sites in the central loop between the sixth and seventh transmembrane domains, which can be phosphorylated under low temperature to increase the transport of glucose and fructose to the vacuole^38^.

Previous studies have demonstrated that sugars increase the expression levels of genes associated with anthocyanin biosynthesis in *Petunia* hybrid^39^ and induce *F3H* expression to increase anthocyanin content in grape berries^40^ In *Arabidopsis*, the sucrose transporter AtSUC1 plays a key role in sucrose-induced anthocyanin accumulation^41^, while in apple, the biosynthetic pathway for UDP sugar contributes to the biosynthesis of cyanidin 3-galactoside in fruit skin^42^. However, the molecular mechanisms underlying the relationship between sugar biosynthetic pathways and anthocyanin accumulation in apple are still unclear.

In this work, we found a monosaccharide transporter, MdTMT1, that localizes to the tonoplast. Overexpression of *MdTMT1* in the *Arabidopsis tmt1* mutant or red-fleshed apple callus increased the contents of glucose and fructose, while MdTMT1 inhibited anthocyanin biosynthesis by decreasing the amounts of UDP-galactose (UDP-gal) and UDP-glucose (UDP-glu). The results of GUS analyses, yeast one-hybrid, chromatin immunoprecipitation-PCR (ChIP-PCR), and electrophoretic mobility shift analyses (EMSA) revealed that MdMYB6 can directly bind to the promoter region of *MdTMT1* and lead to upregulation of gene expression. Overexpression of *MdMYB6* in the calli overexpressing *MdTMT1* increased the expression of *MdTMT1*, which led to reduced UDP-glu and UDP-gal contents. In addition, MdMYB6 was shown to bind the promoters of *MdGSTF12* and *MdANS* and inhibit their expression. In summary, MdMYB6 suppresses anthocyanin biosynthesis both by directly inhibiting *MdGSTF12* and *MdANS* and by reducing UDP-glu and UDP-gal contents by regulating MdTMT1.

## Results

### Contents of soluble sugars and anthocyanin and the expression levels of transcription factors associated with anthocyanin biosynthesis in ‘Hongcui 1’ apple during fruit development

**Fig. 1:**
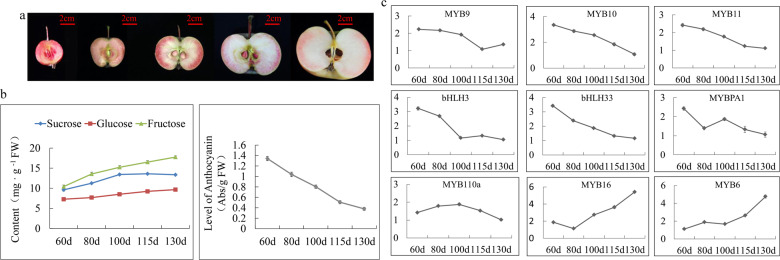
The contents of soluble sugars and anthocyanin and the relative expression levels of genes encoding transcription factors involved in the biosynthesis of anthocyanin in ‘Hongcui 1’ apple during fruit development. **a** Flesh cross-sectional view of ‘Hongcui 1’ apple during development. **b** The contents of soluble sugars and anthocyanin. **c** The relative transcript levels of genes encoding transcription factors involved in the biosynthesis of anthocyanin.

To examine the anthocyanin levels, sugar contents and transcription factor expression levels during ‘Hongcui 1’ apple maturation, we took cross sections of the apple flesh throughout development. Overall, the flesh color became lighter and the anthocyanin content gradually decreased over the course of development (Fig. 1a, b), while the transcript levels of genes encoding regulatory factors that positively affect anthocyanin biosynthesis, such as *MdMYB9*, *MdMYB10*, *MdMYB11*, *MdMYBPA1*, *MdMYB110a*, *MdbHLH3* and *MdbHLH33*, were also reduced during fruit development. Moreover, the transcript levels of genes encoding negative regulatory factors, including *MdMYB6* and *MdMYB16*, increased during fruit development (Fig. 1c). The contents of soluble sugars in the flesh of ‘Hongcui 1’ apple, including glucose, fructose and sucrose, increased during development (Fig. 1b). Although a previous study detected a positive correlation between the coloration of ‘Fuji’ apple skin and the concentration of soluble sugars in apple flesh^43^, we considered it unintuitive to compare apple skin with apple flesh. Our results indicated that there might be a negative correlation between anthocyanin content and soluble sugar content in ‘Hongcui 1’ apple flesh.

### Quantitative proteomic comparison between calli expressing green fluorescent protein (GFP) and calli expressing MdMYB6-GFP

**Fig. 2:**
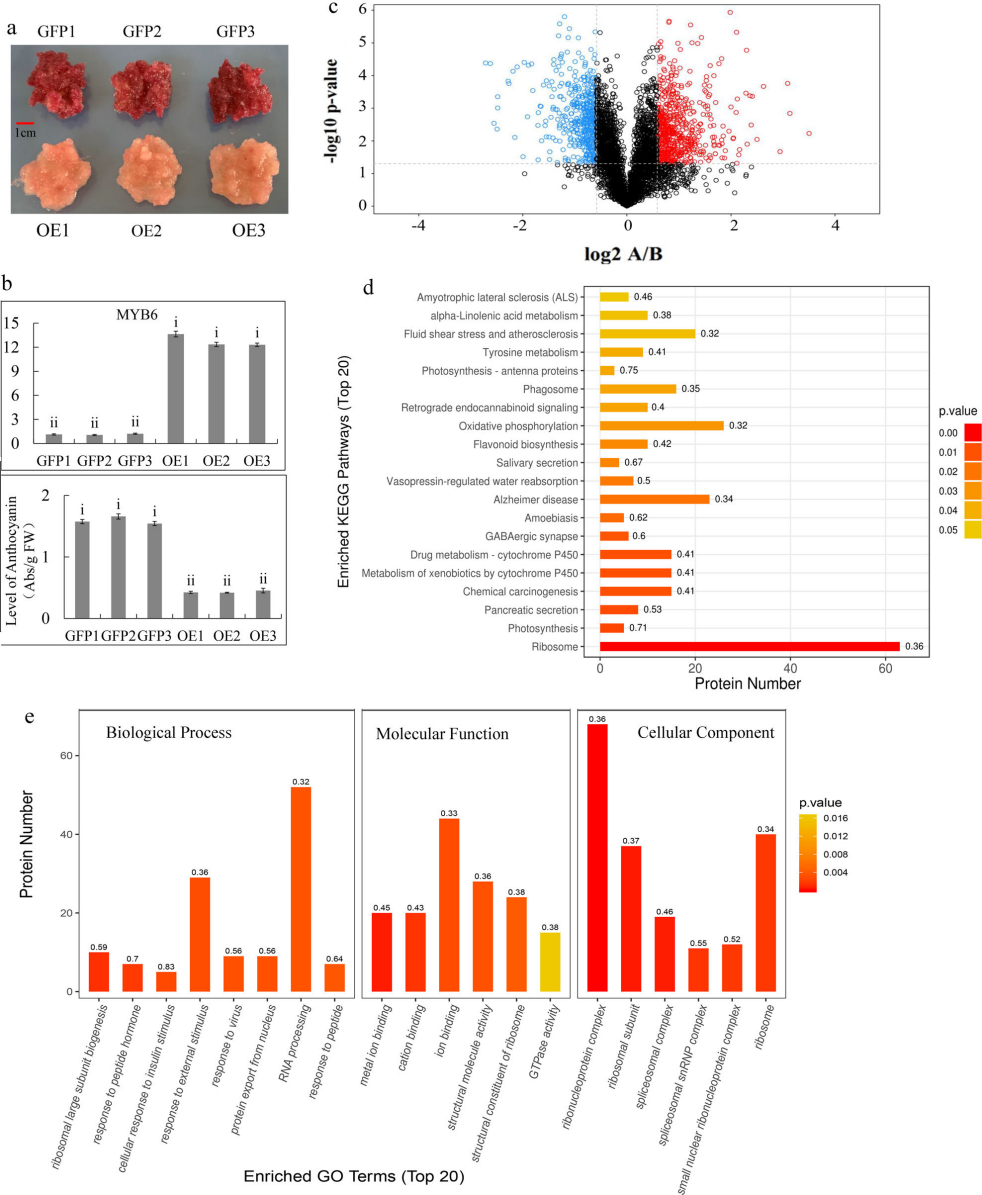
Analysis of proteins that were differentially expressed between the callus expressing GFP (group A) and the callus expressing MdMYB6-GFP (group B). **a** Red-fleshed callus and callus overexpressing *MdMYB6*. **b** Transcript levels of *MdMYB6* and anthocyanin content. **c** Volcano plot of all differentially expressed proteins. The *x*-axis represents significant differences between A and B after log2 transformation. The *y*-axis represents significant differences in the *P* value after log10 transformation. Red dots represent upregulated proteins, while blue dots represent downregulated proteins in A vs. B. **d** KEGG pathway enrichment analysis of the proteins that were differentially expressed between A and B. The *y*-axis represents significantly enriched KEGG pathways, while the *x*-axis indicates the number of differentially expressed proteins in the KEGG pathways. The color gradient represents the *P* value, where red denotes small *p* values and more significant KEGG pathway enrichment. **e** Enriched GO terms of proteins that were differentially expressed between A and B. The *x*-axis represents the enriched GO functional classification, which is divided into three categories: biological process, molecular function and cellular component, while the *y*-axis indicates the number of differentially expressed proteins under each functional classification. The color gradient represents the *P* value, where red denotes small *P* values and more significant GO functional classification enrichment.

To investigate the function of *MdMYB6* in regulating sugar and anthocyanin levels, we generated *MdMYB6* overexpression lines. In the red-fleshed calli, the overexpression of *MdMYB6* led to a change in color from red to pink, indicating reduced anthocyanin content (Fig. 2a,b). To identify differentially expressed proteins involved in this color change, we compared the proteomes of the calli overexpressing GFP (control) and the calli overexpressing MdMYB6-GFP using the tandem mass tags (TMT) quantitative proteomics technique (Zhongke New Life Co., Shanghai, China). In total, 4865 proteins were identified in the self-built library. According to the criteria for differential expression (>1.5-fold differences in expression levels between the two libraries, and *P* values < 0.05), we found 609 upregulated proteins and 477 downregulated proteins in the control versus the MdMYB6-GFP overexpression line (Fig. 2c).

**Fig. 3:**
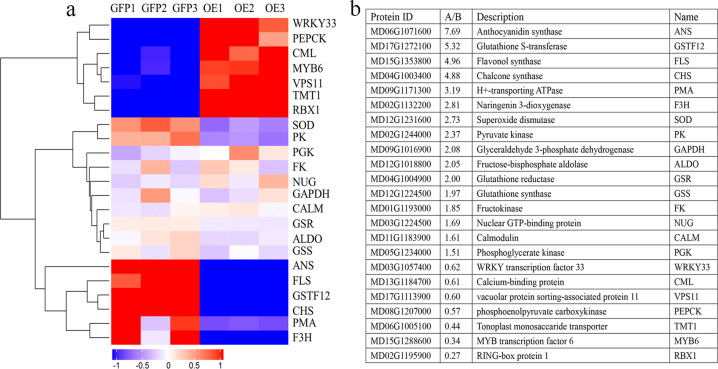
Screening of 23 differentially expressed proteins and the relative protein expression levels between A (callus expressing GFP) and B (callus expressing MdMYB6-GFP). **a** Heatmap-cluster analysis of the 23 proteins that were differentially expressed between A and B. Boxes denote protein expression levels after log2 conversion, while colors indicate the Z-score values of normalized protein expression. **b** Descriptions of the 23 screened proteins and their relative protein expression levels in A and B.

The GO functional analysis of the proteins that were differentially expressed between the control and the MdMYB6-GFP overexpression line found that several important biological terms, such as biological process, molecular function and cellular component, were enriched (Fig. 2e). The results of KEGG pathway analysis showed that significant differences had occurred in the ribosome, photosynthesis, pancreatic secretion and other pathways (Fig. 2d). In the calli overexpressing *MdMYB6-GFP*, the upregulated proteins included MdMYB6, MdTMT1, MdWRKY33 and MdVPS11, and the downregulated proteins included MdANS, MdFLS, MdGSTF12, MdCHS, and MdF3H (Fig. 3a, b).

### Interactions between MdMYB6 and the promoters of *MdANS* and *MdGSTF12*

**Fig. 4:**
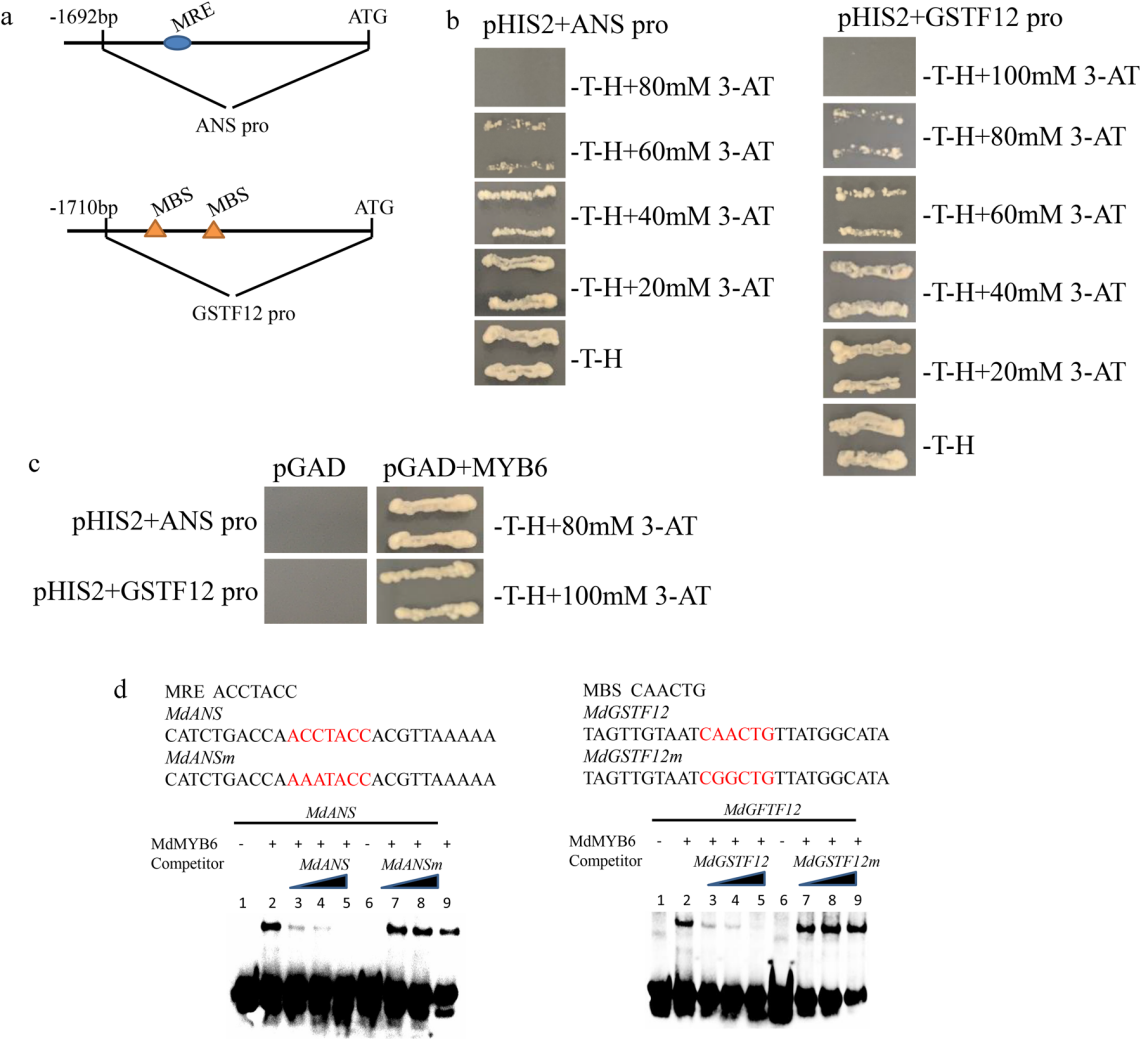
Analyses of the interactions between MdMYB6 and the promoters of *MdANS* and *MdGSTF12*. **a** The 1692-bp sequence of the *MdANS* promoter and the 1710-bp sequence of the *MdGSTF12* promoter. **b** Screening of different concentrations of 3-AT to determine the optimum reporter gene inhibition. **c** Yeast one-hybrid analysis of the interactions between the MdMYB6 protein and the promoters of *MdANS* and *MdGSTF12*. **d** Electrophoretic mobility shift analysis of the interactions between the MdMYB6 protein and the promoters of *MdANS* and *MdGSTF12*. Lane 1 includes the labeled DNA probes with no protein added, while lane 2 includes protein as well as the labeled DNA probes without any competitor. Unlabeled DNA probes were added in increasing amounts (25×, 50×, and 100×) in lanes 3, 4, and 5. Unlabeled mutant DNA probes (lanes 7, 8, and 9) were added as competitors. In lane 6, labeled mutant DNA probes and protein were added but with no competitor.

The results of the proteomic analysis indicated that MdANS and MdGSTF12, which are key enzymes involved in anthocyanin biosynthesis, were downregulated in the calli overexpressing *MdMYB6-GFP*^5,27^. Therefore, we isolated the 1692-bp promoter of *MdANS* and the 1710-bp *MdGSTF12* promoter sequence to identify target DNA binding site(s) of MdMYB6 using yeast one-hybrid (Y1H) assays (Fig. 4a). Recombinant pHIS2 plasmids containing the *MdANS* and *MdGSTF12* promoters were transformed into Y187 yeast. Each strain was then cultivated in –tryptophan–histidine (–T–H) selective media containing a series of different 3-amino-1,2,4-triazole (3-AT) concentrations, which resulted in a reduction of yeast growth as the 3-AT concentration increased.

The yeast strain containing the pHIS2-MdANSpro plasmid was not able to grow when using –T–H selective medium containing 80 mM 3-AT, while the yeast strain with the pHIS2-MdGSTF12pro plasmid could not grow on the –T–H selective medium with 100 mM 3-AT (Fig. 4b). Thus, 80 mM 3-AT could reduce the expression of the HIS3 reporter when its expression was controlled by the *MdANS* promoter, and 100 mM 3-AT could inhibit the expression of the HIS3 reporter gene when it was placed under the control of the *MdGSTF12* promoter. Next, various combinations of pGADT7-MdMYB6 with pHIS2-MdANSpro and pHIS2-MdGSTF12pro were cotransformed into Y187 yeast. The yeast strain containing both pGADT7-MdMYB6 and pHIS2-MdANSpro grew on -tryptophan–histidine–leucine (–T–H–L) selective media supplemented with 80 mM 3-AT, while the yeast strain containing both pGADT7-MdMYB6 and pHIS2-MdGSTF12pro grew on –T–H–L selective media that contained 100 mM 3-AT. The yeast strains containing combinations of pGADT7 plasmids driven by pHIS2-MdANSpro and pHIS2-MdGSTF12pro were unable to grow on any of these selective media (Fig. 4c).

Using tools in the PlantCARE database, we found one MYB recognition element (MRE) that was present in the promoter of *MdANS* and two MYB binding site (MBS) elements in the *MdGSTF12* promoter. Therefore, we used electrophoretic mobility shift assays (EMSAs) to analyze the interaction between the MdMYB6 protein and these elements. In the EMSAs, the mixture of MdMYB6 protein and biotin-labeled probes containing the identified MRE yielded a dense band, which disappeared with increasing concentrations of the probe without the biotin label. The mixture of MdMYB6 protein and a biotin-labeled probe containing a mutated MRE did not yield a band in this analysis (Fig. 4d). Similar results were observed in an EMSA with a mixture of MdMYB6 protein and a biotin-labeled probe containing the MBS element (Fig. 4d). Taken together, the results of the EMSAs showed that MdMYB6 can bind to the promoters of both *MdANS* and *MdGSTF12*.

### Protein sequence analysis, phylogenetic tree construction, and subcellular localization of MdTMT1

**Fig. 5:**
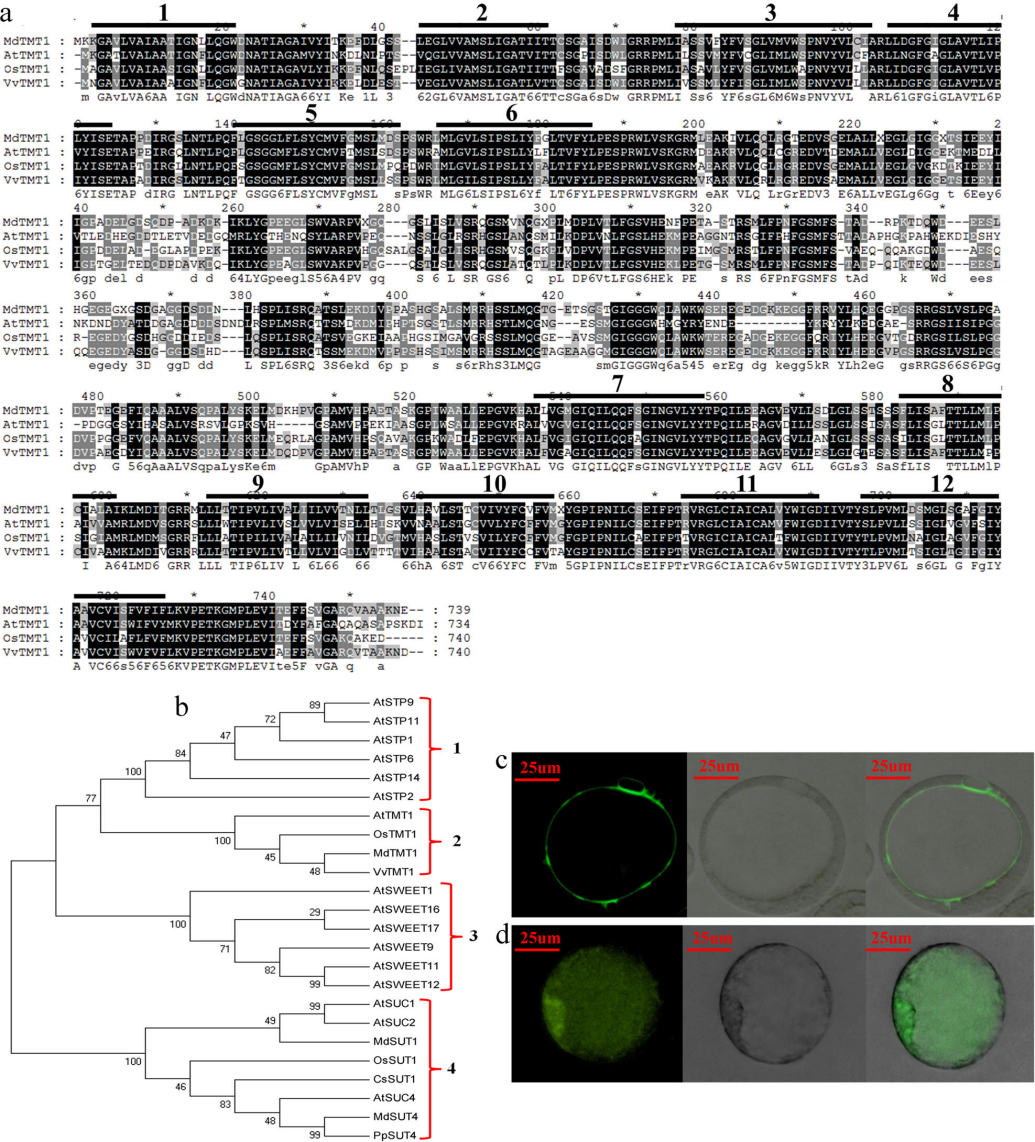
MdTMT1 protein sequence, phylogenetic tree, and subcellular localization analysis. **a** Comparison of the predicted protein structures of MdTMT1, AtTMT1, CsTMT1, and VvTMT1. **b** Phylogenetic tree of proteins related to sugar transport. (**c**) Subcellular localization analysis of MdMYB6-GFP in apple callus protoplasts. The first panel shows a GFP fluorescence image, the second panel shows a bright field image, and the last panel shows the merged GFP fluorescence and bright field image. **d** Subcellular localization analysis of GFP in apple callus protoplasts. The details of the 3 panels are the same as in **c**.

Apple tonoplast monosaccharide transporter 1 (TMT1), which was differentially expressed between the calli overexpressing GFP and the calli overexpressing MdMYB6-GFP, was subjected to KEGG pathway and functional annotation analyses. MdTMT1 has 739 amino acid residues and shows 63.88% similarity to AtTMT1, 71.18% similarity to OsTMT1, and 80.32% similarity to VvTMT1 at the amino acid sequence level (Fig. 5a). We also detected strong similarities of the transmembrane domains among these four TMT proteins. The MdTMT1 protein was predicted to contain 12 transmembrane domains and a large central hydrophilic loop that spans the region that connects transmembrane domains 6 to 7 (Fig. 5a). This domain architecture was highly similar to the structures of AtTMT1, OsTMT1 and VvTMT1^34^.

Next, we constructed a phylogenetic tree based on the protein sequences of MdTMT1 and other sugar transporters. This tree resulted in four groups: members of the STP family, members of the TMT family (including MdTMT1), members of the SWEET family, and members of the SUT/SUC family (Fig. 5b).

In the subcellular localization analyses, the fluorescence of GFP fused with MdTMT1 was only observed in the tonoplast of protoplasts (Fig. 5c), while that of the unfused GFP was detected throughout the whole protoplast (Fig. 5d). These results clearly indicate that MdTMT1 is localized to the tonoplast.

### MdTMT1 overexpression increased the glucose and fructose contents in *Arabidopsis* and the red-fleshed apple calli

**Fig. 6:**
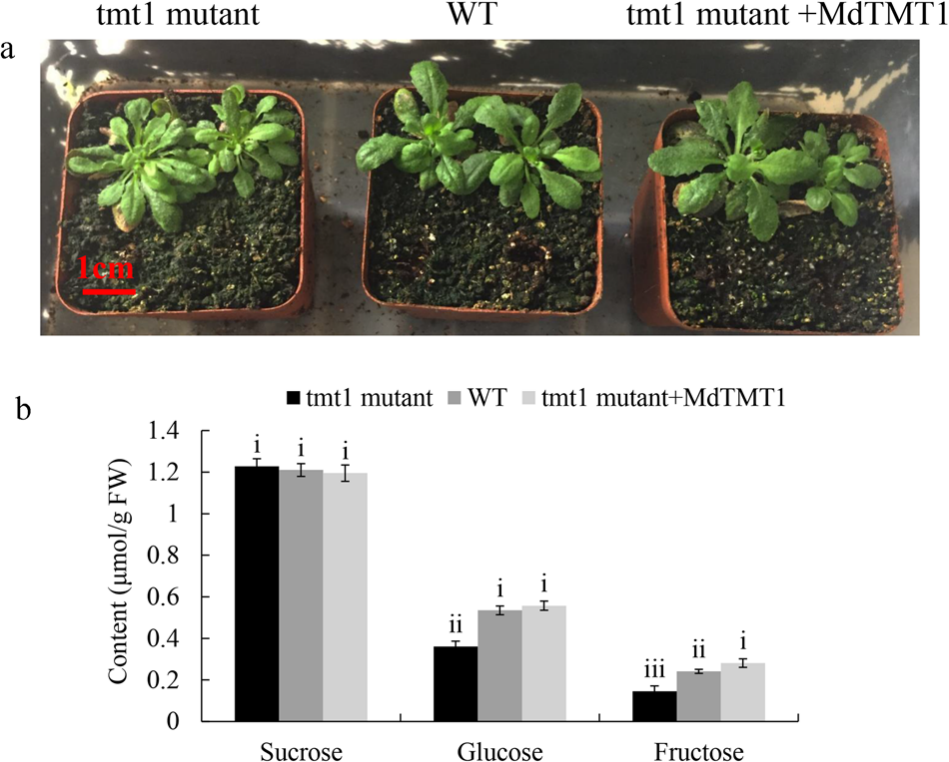
Functional characterization of MdTMT1 by overexpression in the *Arabidopsis TMT1* mutant (*tmt1*). Significance tests are labeled i, ii, iii, and iv. Different letters above columns denote significant differences (*P* < 0.01). **a** Forty-day-old plants of wild-type *Arabidopsis* (WT), *TMT1* mutant (*tmt1*) and *tmt1* overexpressing *MdTMT1* (*tmt1* mutant+*MdTMT1*). **b** Contents of sucrose, glucose, and fructose in the above three *Arabidopsis* lines.

**Fig. 7:**
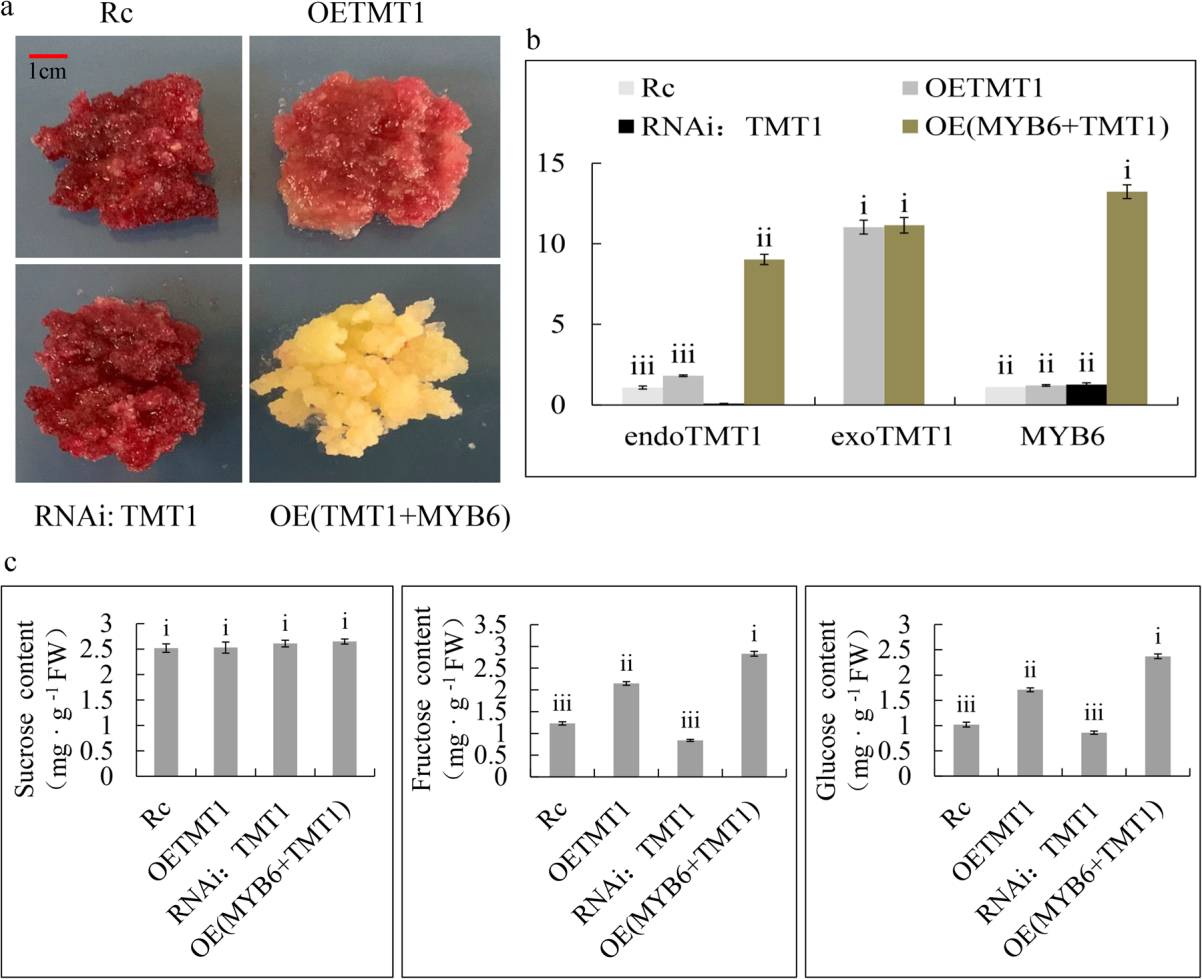
Functional characterization of MdTMT1 by overexpression in red-fleshed apple callus. Significance tests are shown as i, ii, iii, and iv. Different letters above the columns denote significant differences (*P* < 0.01). **a** Red-fleshed apple callus (Rc), red-fleshed apple callus overexpressing *MdTMT1* (OETMT1), red-fleshed apple callus with silenced *MdTMT1* (RNAi: TMT1) and red-fleshed apple callus co-overexpressing *MdMYB6* and *MdTMT1* (OE(TMT1 + MYB6)). **b** Transcript levels of *MdMYB6* and *MdTMT1* in the above four calli. Endogenous *MdTMT1* (endoTMT1), exogenous *MdTMT1* (exoTMT1). **c** Contents of sucrose, glucose, and fructose in the four calli.

To test the function of *MdTMT1*, we determined the contents of sucrose, glucose, and fructose in 40-day-old plants of the wild-type *Arabidopsis* (WT), the TMT1 mutant of *Arabidopsis* (*tmt1*), and *tmt1* overexpressing *MdTMT1* (*tmt1* + MdTMT1) (Fig. 6a). Although there was no difference in the sucrose content among these three lines, the contents of glucose and fructose were lower in the *tmt1* plants than in the WT plants. Overexpression of *MdTMT1* in *tmt1* restored the contents of glucose and fructose to some extent (Fig. 6b). Overexpressing *MdTMT1* in red-fleshed calli increased the contents of glucose and fructose but not that of sucrose. When *MdTMT1* was silenced in the red-fleshed calli using RNAi, the contents of glucose and fructose were reduced (Fig. 7a, c). Finally, overexpressing *MdMYB6* in the *MdTMT1*-overexpressing calli increased the transcript level of *MdTMT1* (Fig. 7a, b).

### MdMYB6 bound to and activated the *MdTMT1* promoter

**Fig. 8:**
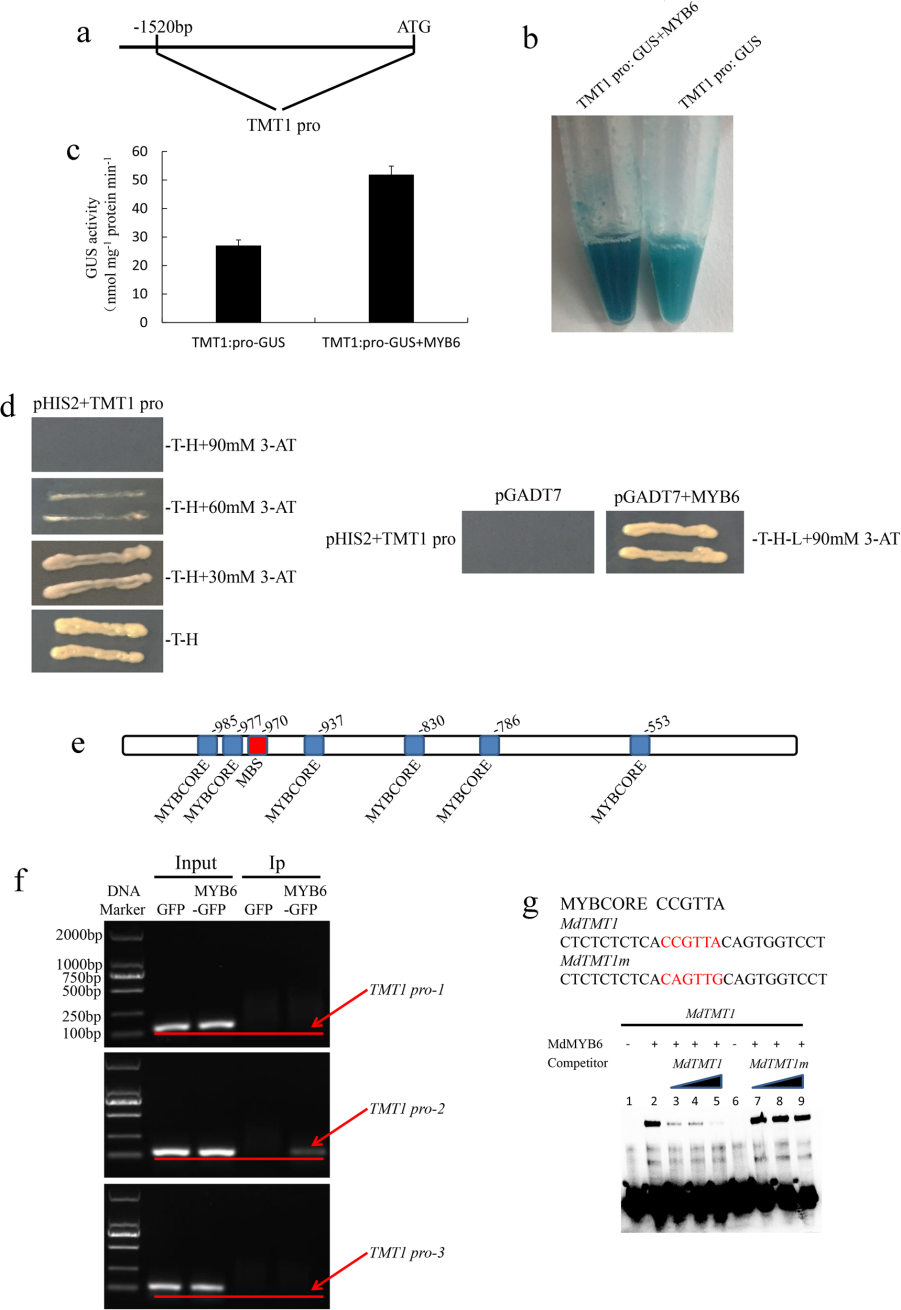
Analyses of the interaction between the *MdTMT1* promoter and MdMYB6 protein. **a** The 1520-bp sequence of the *MdTMT1* promoter. **b** GUS histochemical staining of a callus harboring a plasmid containing the *MdTMT1* promoter and GUS reporter gene (TMT1 pro: GUS) and a callus overexpressing MdMYB6 harboring the TMT1 pro:GUS vector (TMT1 pro: GUS + MYB6). **c** Analysis of GUS activity. **d** Yeast one-hybrid analysis of the interaction between the MdYB6 protein and the *MdTMT1* promoter. **e** Predicted elements bound by MdMYB6 in the *MdTMT1* promoter. **f** ChIP-PCR analyses of the interaction between the *MdTMT1* promoter fragments and the MdMYB6 protein. MdMYB6-DNA complexes were then coimmunoprecipitated from the calli overexpressing MdMYB6-GFP via a GFP antibody. **g** EMSA analysis of the interaction between the *MdTMT1* promoter and MdMYB6 protein. Lane 1 included labeled DNA probes with no added protein, while lane 2 included labeled DNA probes with no competitor or protein. Unlabeled DNA probes were added in increasing amounts (25×, 50×, and 100×) in lanes 3, 4, and 5. Unlabeled mutant DNA probes (lanes 7, 8, and 9) were added as competitors. Lane 6 included labeled mutant DNA probes and protein but without competitor.

Next, we utilized the 1520-bp sequence of the *MdTMT1* promoter for the GUS reporter assay and Y1H analysis (Fig. 8a). In the GUS histochemical staining analyses, the callus with both the *MdTMT1* promoter and MdMYB6 was more deeply stained than the callus with only the *MdTMT1* promoter (Fig. 8b). Consistent with this result, GUS activity was higher in the calli with both the *MdTMT1* promoter and MdMYB6 than in the calli with only the *MdTMT1* promoter (Fig. 8c). In the Y1H assay, MdMYB6 could bind to the promoter of *MdTMT1* (Fig. 8d).

Using tools from the PlantCARE database, we found six MYBCORE and one MBS elements within the 1520-bp sequence of the *MdTMT1* promoter (Fig. 8e). The sequences from −492 to −688, −700 to −874, and −908 to −1084 were termed *TMT1* pro-1, *TMT1* pro-2, and *TMT1* pro-3, respectively. *TMT1* pro-1 contained one MYBCORE element, *TMT1* pro-2 contained two MYBCORE elements, and *TMT1* pro-3 contained one MBS and three MYBCORE elements. These three promoter regions were amplified for ChIP-PCR analyses. As expected, the input of GFP and MdMYB6-GFP all produced bands in analyses for the three promoter regions, the IP of GFP alone produced no band and that of MdMYB6-GFP produced a band corresponding to *TMT1* pro-2 (Fig. 3b). These results indicate that MdMYB6 can bind to the sequence from −700 to −874 in the *MdTMT1* promoter (Fig. 8f). In the EMSAs, MdMYB6 could bind to only one MYBCORE element (CCGTTA) in the sequence from −830 to −835 (Fig. 8g). Taken together, these analyses showed that MdMYB6 binds to the MYBCORE element in the promoter of *MdTMT1* and increases its activity.

### MdMYB6 and MdTMT1 may be involved in anthocyanin metabolism

**Fig. 9:**
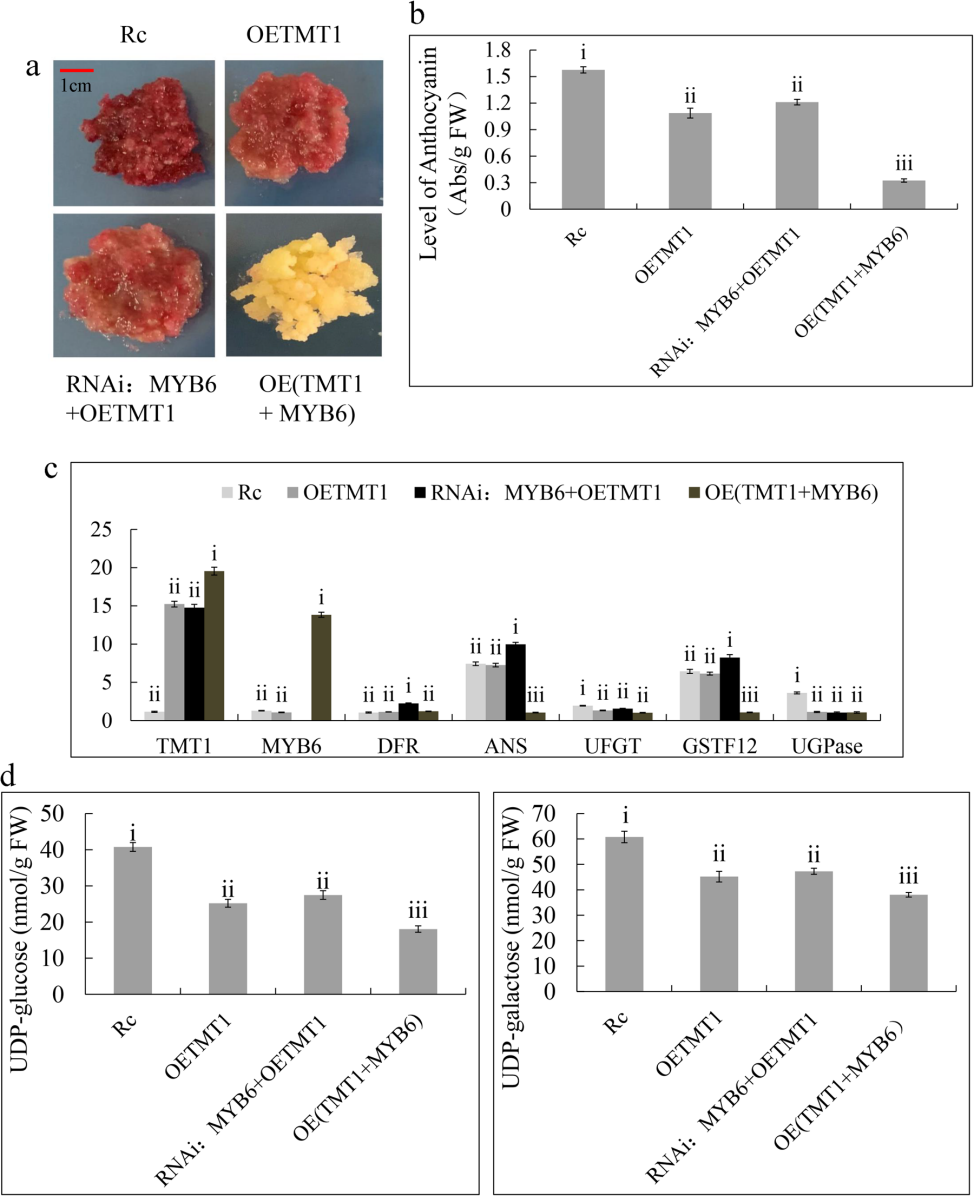
Effects of MdMYB6 and MdTMT1 on anthocyanin biosynthesis in red-fleshed apple callus. Significance tests are labeled i, ii, iii, and iv, with different letters above columns denoting significant differences (*P* < 0.01). **a** Red-fleshed apple callus (Rc), red-fleshed apple callus overexpressing *MdTMT1* (OETMT1), red-fleshed apple callus with silenced *MdMYB6* and overexpression of *MdTMT1* (RNAi: MYB6 + OETMT1), red-fleshed apple callus co-overexpressing *MdMYB6* and *MdTMT1* (OE(TMT1 + MYB6)). **b** Anthocyanin levels in all four calli. **c** Transcript levels of key genes in the four transfected calli. **d** Contents of UDP-glu and UDP-gal in the four calli.

TMT proteins are transmembrane sugar transporters^34,37^, but overexpression of *MdTMT1* in red-fleshed apple callus changed the color from red to light red, indicating that MdTMT1 can also affect anthocyanin content (Fig. 9a, b). In our analyses, MdMYB6 inhibited anthocyanin biosynthesis (Fig. 2a, b) and affected the expression of *MdTMT1* (Figs. 3a, b and 7b). To rule out the possibility of an inhibitory effect of endogenous *MdMYB6*, we used RNA interference (RNAi) technology to silence *MdMYB6* expression.

Overexpression of *MdTMT1* in the calli with silenced *MdMYB6* still led to a change in the color of the calli from red to light red (Fig. 9a), which was associated with reduced anthocyanin levels (Fig. 9b). This finding indicates that MdTMT1 functioned independently in inhibiting anthocyanin accumulation. Overexpression of *MdMYB6* in the red-fleshed calli led to reduced expression of *MdANS* and *MdGSTF12* and increased expression of *MdTMT1* (Fig. 9c). Overexpression of *MdTMT1* in the red-fleshed callus decreased the expression of *MdUGPase* (Fig. 9c) and the contents of UDP-glu and UDP-gal (Fig. 9d), while overexpression of *MdMYB6* in the callus overexpressing *MdTMT1* decreased the contents of UDP-glu and UDP-gal (Fig. 9d).

## Discussion

### MdMYB6 specifically binds to the promoters of *MdANS* and *MdGSTF12* and inhibits anthocyanin biosynthesis

MYB transcription factors (TFs) play critical roles in anthocyanin metabolism by controlling the expression levels of several genes encoding key structural enzymes involved in anthocyanin biosynthesis^44^. Previous studies have shown that the promoter of *MdANS* can be stimulated by the binding of both MdMYB9 and MdMYB11^25^. Several MYB proteins have been shown to recognize and bind to MYBCORE, MRE, and MBS elements in gene promoters^45–47^. In apple, we found that MdMYBPA1 binds to the MRE in the promoter of *MdANS* and to the MBS element in the promoter of *MdUFGT*, resulting in increased transcription and higher anthocyanin content^47^.

There are two types of MYB TFs that have been shown to reduce anthocyanin biosynthesis, one of which is dependent on its own EAR inhibitory sequence, while the other is independent^19,48–50^. In addition, there are two mechanisms by which MYB repressors control anthocyanin biosynthesis: they can act upon MBW complexes (FaMYB1^51^ and MdMYB15L^52^) or directly bind to the promoters of target genes (AtMYB4^53^ and MdMYB16^19^).

In our study, overexpression of *MdMYB6* in the callus of red-fleshed apple decreased the anthocyanin content (Fig. 2b) and downregulated *MdANS* (Figs. 3a, b and 9c). The Y1H assay revealed that MdMYB6 can bind to the *MdANS promoter* (Fig. 4a, b, c), while the EMSA analysis showed that MdMYB6 can recognize and bind to the MRE in the promoter of *MdANS* (Fig. 4d). This finding was consistent with a previous study that showed that MdMYB6 could suppress the expression of *AtANS* in transgenic *Arabidopsis*^18^. Recent studies have shown that anthocyanins are synthesized in the cytoplasm and need to be moved to the vacuole for storage. Glutathione S-transferase (GST) was reported to participate in the transport of anthocyanin, such as transparent testa 19 (*AtTT19*) in *Arabidopsis*^26^, *LcGST4* in litchi^54^ and *MdGSTF6* in apple^27^. In this study, the comparative proteomic analysis between the calli overexpressing GFP and those overexpressing MdMYB6-GFP revealed that the protein level of MdGSTF12 was downregulated in the calli overexpressing MdMYB6-GFP (Fig. 3). Overexpression of *MdMYB6* in the red-fleshed callus reduced the transcript level of *MdGSTF12* (Fig. 9c), while the Y1H and EMSA analyses demonstrated that MdMYB6 can directly bind to the MBS element in the *MdGSTF12* promoter (Fig. 4). Together, these results suggest that MdMYB6 inhibits anthocyanin biosynthesis by binding to the promoters of both *MdANS* and *MdGSTF12*, resulting in reduced transcriptional activity.

### MdMYB6 promotes the expression of *MdTMT1*, which transports glucose and fructose across the tonoplast

The vacuole is the largest subcellular organelle in plant cells, accounting for more than 90% of the total cell volume. It is the storage site for most metabolites and soluble sugars in plant cells^36,55^. Recent studies on sugar transporters have revealed some of the mechanisms of sugar transport across the tonoplast. For example, tonoplast-localized TMT-like monosaccharide transporters mediate the transport of monosaccharides into the vacuole via a reverse transport mechanism coupled with protons^34,37,56^. The TMT proteins generally have twelve transmembrane domains and a long loop connecting the sixth and seventh transmembrane domains^38^. In *Arabidopsis*, a subcellular localization assay showed that AtTMT1 is localized at the tonoplast. The contents of glucose and fructose in leaves were found to be significantly lower in the *AtTMT1* mutant plants than in the wild-type plants, indicating that AtTMT1 mediates the transport of monosaccharides into the vacuole^34^. In grape, *VvTMT1* is significantly expressed in the fruit and is associated with glucose and fructose accumulation during fruit development^57^. We found that MdTMT1 has the same transmembrane domain structure as its homologs in grape and *Arabidopsis* (Fig. 5a) and is also localized to the tonoplast (Fig. 5c). Overexpression of MdTMT1 in the *tmt1* mutant of *Arabidopsis* restored the contents of glucose and fructose in leaves (Fig. 6), with similar results observed in apple callus (Fig. 7). Interestingly, silencing of *MdTMT1* in the red-fleshed calli reduced the contents of glucose and fructose but not that of sucrose (Fig. 7c).

A comparative proteomic analysis between the calli overexpressing GFP and those overexpressing MdMYB6-GFP revealed a significant increase in the MdTMT1 protein levels in the calli overexpressing MdMYB6-GFP (Fig. 3). GUS reporter analysis, Y1H, ChIP-PCR and EMSA showed that MdMYB6 binds to the MYBCORE element of the *MdTMT1* promoter, resulting in increased expression of *MdTMT1* (Fig. 8d–g). The transcript level of *MdTMT1* and the contents of glucose and fructose were higher in the calli co-overexpressing *MdMYB6* and *MdTMT1* than in the calli overexpressing *MdTMT1* alone (Fig. 7b, c). Together, these results suggest that MdMYB6 promotes the expression of *MdTMT1*, which transports glucose and fructose into the vacuole, thereby increasing their contents in the callus. However, MdMYB6 was a repressor, and there was no mechanism to show how it could act as an activator to promote the expression of *MdTMT1*, which might be why MdMYB6 inhibited a repressor of MdTMT1 that would indirectly lead to activation.

### MdTMT1 may decrease anthocyanin content by reducing the levels of substrates for anthocyanin biosynthesis

Anthocyanidins are synthesized from leucoanthocyanidin in a reaction catalyzed by anthocyanidin synthase (ANS). Although these intermediate compounds are unstable, UDP-sugar:flavonoid 3-O-glycosyltransferase (UFGT) can rapidly catalyze the conversion of anthocyanidins to anthocyanins by adding a UDPG sugar. In some plants, such as *Antirrhinum majus*, *Gentiana triflora*, *Perilla frutescens*, and *Vitis vinifera*, the preferred acceptor is anthocyanidin, and the donor is generally UDP-glu^58^, making UDP-glu content an extremely important factor for anthocyanin biosynthesis. The substrate UDP-gal was found to be positively correlated with anthocyanin content^42^ and is thought to be synthesized from UDP-glu.

Although many studies have focused on the regulation of structural genes that are required for anthocyanin biosynthesis at the transcriptional and protein modification levels, few studies have focused on substrates for anthocyanin synthesis. In our study, overexpression of *MdTMT1* in the red-fleshed callus reduced the contents of UDP-glu and UDP-gal (Fig. 9d), and decreased the expression of *UGPase*, which encodes the enzyme responsible for converting glucose 1-phosphate to UDP-glu (Fig. 9c). Overexpression of *MdMYB6* in the calli overexpressing *MdTMT1* increased the expression of *MdTMT1*, which led to reduced UDP-glu and UDP-gal contents (Fig. 9d). This change resulted in decreased contents of UDP-glu and UDP-gal (Fig. 9d) and consequently reduced anthocyanin content (Fig. 9a, b). This result indicated that MdMYB6 could inhibit anthocyanin biosynthesis by regulating *MdTMT1* to decrease the contents of UDP-glu and UDP-gal. Overexpression of *MdTMT1* in the calli with silenced *MdMYB6* also led to decreased anthocyanin content (Fig. 9). These results indicate that the inhibitory effect of *MdTMT1* on anthocyanin content is not affected by the presence of endogenous *MdMYB6*. The mechanism by which MdTMT1 decreases the contents of UDP-glu and UDP-gal is unknown and should be explored in further research.

**Fig. 10:**
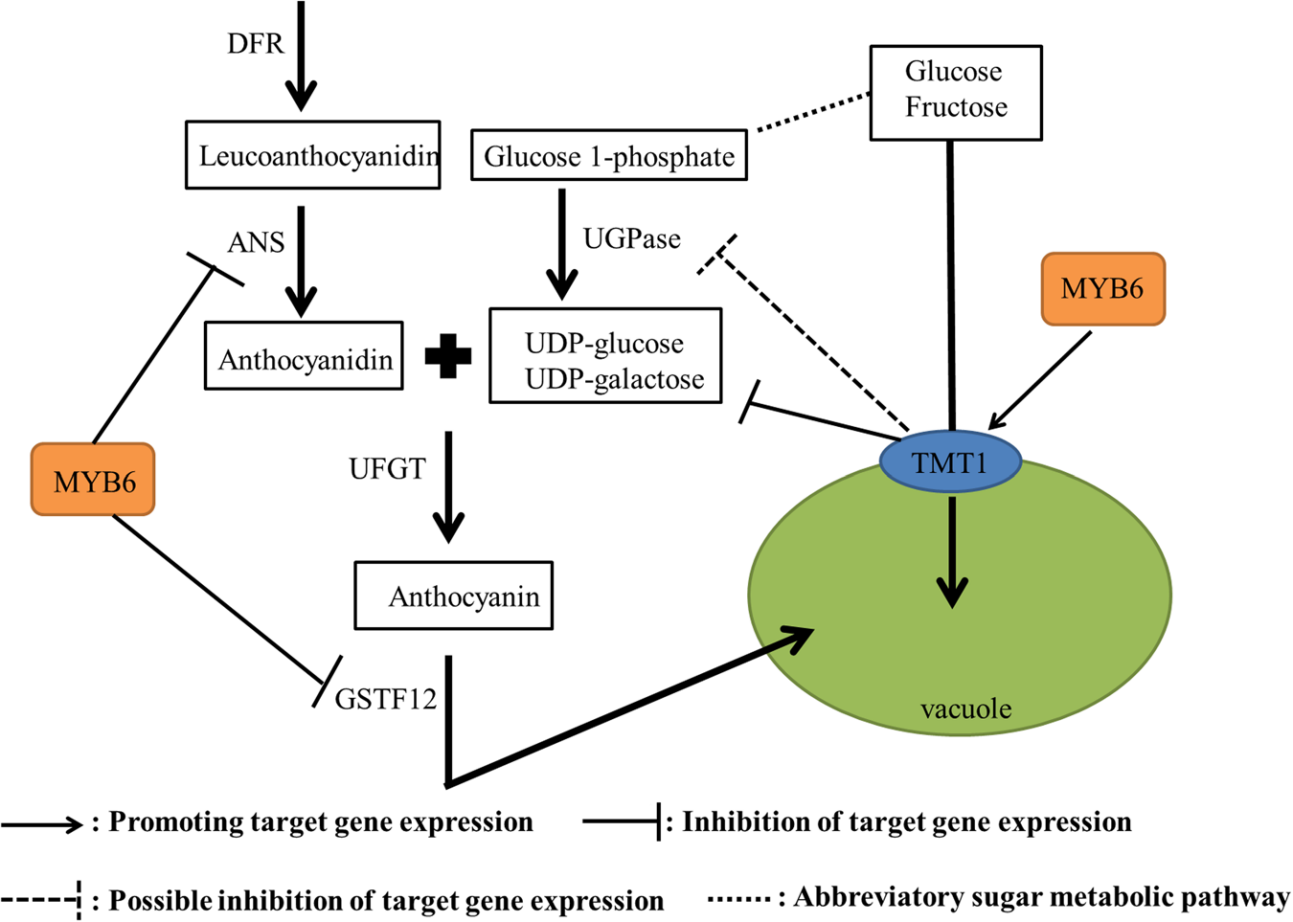
Putative regulatory network of anthocyanin biosynthesis and monosaccharide transport across the tonoplast controlled by MdMYB6 and MdTMT1.

On the basis of the above results, we propose a regulatory network of anthocyanin biosynthesis and the transport of monosaccharides across the tonoplast, which are controlled by MdMYB6 and MdTMT1 (Fig. 10). As shown in our analyses, MdMYB6 can directly bind to the promoters of *MdANS* and *MdGSTF12* and reduce their expression, thereby inhibiting the biosynthesis of anthocyanin. Furthermore, MdTMT1 decreases the contents of UDP-glu and UDP-gal, leading to a reduction in anthocyanin content. MdTMT1, which is located at the tonoplast, also increases the contents of glucose and fructose in plant cells, while MdMYB6 can bind to the *MdTMT1* promoter and drive increased *MdTMT1* expression. These results significantly expand the current understanding of the relationship between sugar levels and anthocyanin contents in apple.

## Materials and Methods

### Plant materials and processing

‘Purple 3’ and ‘Hongcui 1’ apples were selected from first-generation hybrids created by crossing *Malus sieversii* f. *niedzwetzkyana* with *Malus × domestica* cv. Fuji. The flesh of individual ‘Hongcui 1’ apples was collected five times during fruit development at 60, 80, 100, 115, and 130 days after flowering. The collected tissue was then frozen in liquid nitrogen before storage at −80 °C. Red-fleshed apple callus was created from the young leaves of the ‘Purple 3’ apples, as described by Ji et al^59^. The red-fleshed callus used for genetic transformation was cultured in Murashige and Skoog medium (MS), which was supplemented with a mixture of 1 mg/L 6-benzylaminopurine and 0.3 mg/L 1-naphthylacetic acid, for 2 weeks. The *Arabidopsis tmt1* mutant (stock code N874344) was purchased from http://arabidopsis.info/.

### Anthocyanin extraction and quantification

Anthocyanin was extracted as previously described by Jin et al.^53^, with slight alterations. One gram of plant tissue was first ground to powder in liquid nitrogen, followed by extraction with a solution of 40 mL of 1% (v/v) HCl-methanol at 4 °C in the dark for 24 h. The resulting solution was then centrifuged at 12,000×*g* for 10 min. An ultraviolet spectrophotometer was then used to determine the supernatant absorbance at 530 nm.

### Extraction of total RNA and qRT-PCR analyses

RNA was extracted via a kit (TransGen Biotech, Beijing, China), as described by Xu et al^19^. The qRT-PCR analyses were carried out on the Bole CFX96 system (Bio-Rad, Hercules, CA, USA) according to the manufacturer’s recommended protocols. Each sample was analyzed in triplicate, with *MdActin* used as the internal reference gene for each quantification. The Ct values were read under default conditions, and the 2^−ΔΔCT^ method was used for data analysis^60^.

### Determination of sucrose, glucose and fructose contents

According to the method of Zhao et al.^61^, 5 g of tissue was ground into a homogenate and transferred to a 30-mL centrifuge tube. After addition of 25 mL of ultrapure water, the mixture was subjected to ultrasonic extraction in an 80 °C water bath for 1 h, cooled, and then centrifuged for 15 min at 10,000×*g*. The supernatant was then filtered into a 50-mL volumetric flask, and the residue was added to 15 mL of ultrapure water for extraction. The supernatant volume was adjusted to 50 mL using ultrapure water. The sample was then filtered through a 0.22-μm filter before being assessed via high-performance liquid chromatography (HPLC). The contents of sucrose, glucose, and fructose were calculated according to standard curves using the peak areas of the samples. HPLC was performed using a Shimadzu RID-10A Refractive Index Detector and a YMC Polyamine II chromatographic column (length × inner diameter: 250 × 4.6 mm^2^, particle diameter: 5 μm); the mobile phase was comprised of acetonitrile:water = 75:25 (v/v); the sensitivity was 4, the injection volume was 10 μL, and the flow rate was 0.8 mL/min at 30 °C.

### Determination of UDP-galactose and UDP-glucose contents

The quantities of UDP-gal and UDP-glu were determined as described by Ban et al.^42^, with slight modifications. Each 5 g sample was ground to powder in liquid nitrogen. One milliliter of 0.5 M perchloric acid was added, and the mixture was kept on ice for 2 min. The samples were then centrifuged at 5000 × *g* for 10 min at 0 °C. The supernatant was taken to pH 6.5 by adding cooled 2.5 M potassium hydroxide in 1.5 M dipotassium hydrogen phosphate. The resulting solution was then centrifuged at 5000×*g* for 5 min at 0 °C to remove the potassium perchlorate precipitate. The supernatant was filtered through a 0.45-μm filter before analysis via HPLC. An L-7100 (Hitachi, Tokyo, Japan) was used for the HPLC pump, with an L-7420 (Hitachi) as the UV-vis detector. A D-7500 (Hitachi) was utilized for the integrator, and an L-7300 (Hitachi) was employed for the column oven. The column was a Supelcosil LC-18T with a 4.6 mm inner diameter × 150 mm (Supelco, Bad Homburg, Germany). Solvent A (100 mM dipotassium hydrogen phosphate, 100 mM potassium dihydrogen phosphate and 8 mM tetrabutylammonium hydrogen sulfate) was adjusted to pH 5.3 via the addition of phosphoric acid. Solvent B consisted of a mixture of 70% solvent A and 30% methanol. The gradient system was as follows: 100% solvent A for 2.5 min, 0–40% solvent B for 14 min, 40–100% solvent B for 1 min, 100% solvent B for 6 min, and 100–0% solvent B for 1 min. The flow rate was set to 1.5 mL/min at a temperature of 30 °C. The eluate was measured at an absorbance of 254 nm, and the peaks were identified based on a comparison to standard retention times.

### Transformation of the red-fleshed apple callus with *MdMYB6* and *MdTMT1*

The red-fleshed apple callus was transformed as described previously^19^. The coding sequences (CDSs) of *MdMYB6* and *MdTMT1* were ligated into the pRI-101 vector containing GFP and the 35S CaMV promoter, respectively, to generate the 35S:MdMYB6-GFP and 35 S:MdTMT1-GFP plasmids (Supplementary Fig. S1A). The CDS of *MdMYB6* was ligated into the pCAMBIA1301 vector, which contained a sequence encoding a His tag as well as the 35S CaMV promoter, to construct the 35S:MdMYB6-His recombinant plasmid (Supplementary Fig. S1E). The recombinant plasmids were transformed into *Agrobacterium tumefaciens* LBA4404. We then infected the red-fleshed calli with the transformed *Agrobacterium* and cocultured them on MS medium in the dark at 24 °C for 48 h. The resulting calli were then moved onto a screening medium containing 250 mg/L carbenicillin and 50 mg/L kanamycin (Solarbio, Beijing, China) for the *MdMYB6-* or *MdTMT1*-overexpressing calli. The screening medium for cultivating the calli cotransfected with *MdMYB6* and *MdTMT1* contained 250 mg/L carbenicillin, 20 mg/L hygromycin and 50 mg/L kanamycin.

### RNA interference to silence *MdMYB6* and *MdTMT1* in the red-fleshed callus

A 317-bp sequence of *MdMYB6* and a 388-bp sequence of *MdTMT1* were used to construct the *MdMYB6* and *MdTMT1* RNAi plasmids. Either *MdMYB6*ps (*MdMYB6* partial sequence) or *MdTMT1*ps (*MdTMT1* partial sequence) was connected to the PFGC-5941 vector containing a chsA intron and the 35 S CaMV promoter in the forward and reverse directions, respectively (see Supplementary Fig. S1F,G). The forward *MdMYB6* and *MdTMT1* partial sequences were named f*MdMYB6*ps and f*MdTMT1*ps, respectively. The reverse *MdMYB6* and *MdTMT1* partial sequences were named r*MdMYB6*ps and r*MdTMT1*ps, respectively. The red-fleshed callus was transformed with these constructs as described above. The calli were screened on medium that contained 20 mg/L hygromycin and 250 mg/L carbenicillin.

### Ectopic expression of MdTMT1 in the *Arabidopsis tmt1* mutant

Ectopic expression was carried out as described by Wang et al.^62^, with slight alterations. The recombinant plasmid containing *MdTMT1* (Supplementary Fig. S1E) was transformed into *A. tumefaciens* GV3101, which was utilized to infect the *Arabidopsis tmt1* mutant. The T1 transgenic *Arabidopsis* plants were then selected by plating onto MS medium containing hygromycin. The hygromycin-resistant seedlings were then moved to soil and grown in a growth chamber (Ningbo-Jiangnan, http://www.nbjnyq.com/). The T2 seeds were then collected and grown as described above, and their soluble sugar contents were determined.

### Protoplast preparation and analysis of subcellular localization

Protoplasts were prepared as described previously by Wang et al.^47^, with slight changes. Two grams of callus containing GFP-tagged OE-MdTMT1 was added to 10 mL of a cell wall lysis solution and then placed under a vacuum for 30 min. This process was followed by incubation in the dark at 24 °C for a total of 12 h. Following incubation, the cell suspension was gently mixed to free the protoplasts before 10 mL of W5 solution was added. Nylon fabric (75 μm pore diameter) was then used to filter the solution, followed by centrifugation at 5000 × *g*. After removal of the supernatant, the protoplasts were suspended in 10 mL of W5 solution and chilled on ice for 30 min. The supernatant was then removed, and the protoplasts were resuspended in 2 mL of MMG solution. An epifluorescence microscope was then used to measure the fluorescence of the protoplasts.

### Yeast one-hybrid (Y1H) analysis

The Y1H assays were performed as described by Xu et al.^19^, with minor alterations. The yeast strain Y187 (Clontech, Palo Alto, CA, USA) was utilized following the manufacturer’s recommended protocols. The CDS of *MdMYB6* was ligated into the pGADT7 vector (Supplementary Fig. S1B), while the promoters of *MdANS*, *MdGSTF12* and *MdTMT1* were ligated into a pHIS2 vector (Supplementary Fig. S1C,D,I). Different combinations of constructs were then cotransformed into Y187, and the interactions were determined on media with optimal 3-AT concentrations that were deficient in Trp, Leu, and His (SD/-Trp-Leu-His).

### GUS staining and activity analyses

The *MdTMT1* promoter was ligated to the pCAMBIA1301 vector, which encodes the GUS protein (described in Supplementary Fig. S1H). The constructed MdTMT1 pro:GUS vector was transferred into the WT and OE-MdMYB6 calli. The expression of GUS was detected by histochemical staining as described by Jefferson et al^63^. Two-week-old calli were submerged in GUS staining buffer, placed under a vacuum for 5 min, and then incubated overnight at 37 °C. The samples were then photographed. For analysis of GUS enzyme activity, 0.5 g of transgenic callus was extracted in 1 mL of GUS extraction buffer. The concentration of the resulting protein sample was then assessed via the Bradford method^64^. GUS activity was then quantified as described previously^22^.

### Electrophoretic mobility shift assays (EMSAs)

The EMSAs were conducted using an EMSA kit (Pierce, Rockford, IL, USA) following the manufacturer’s recommended protocols. The promoter sequences of *MdANS*, *MdGSTF12*, and *MdTMT1* were used to create probes labeled with biotin (Sangon Biotech, Shanghai, China). Fifty nanograms of MdMYB6-His DNA was purified for use in binding reactions, which were carried out in 20-mL reaction mixtures containing 17% glycerol, 0.1 mM ethylenediaminetetraacetic acid, 4 mg poly(dI-dC), 100 mM KCl, 1 mM DTT, 25 mM HEPES-KOH (pH 7.5), competitor DNA (25, 50, or 100 pmol), 1 pmol labeled probe, and 50 ng of purified protein. The reactions were then incubated at room temperature for 30 min. The samples were analyzed by electrophoresis on 6% acrylamide gels containing 3.6% glycerol and 0.5% TBE buffer for 2 h at 4 °C after a prerun in 0.5% TBE buffer at 100 V for 1 h. The DNA was then transferred onto nylon membranes, and the signal was detected by chemiluminescent nucleic acid detection.

### Chromatin immunoprecipitation-PCR (ChIP-PCR) analyses

The ChIP assays were carried out as described by He et al.^65^, with slight changes. A ChIP Kit (Upstate, Lake Placid, NY, USA) was used for crosslinking, removal of crosslinking, immunoprecipitation with a GFP antibody (Abmart, Shanghai, China) and elution. The quantity of the immunoprecipitated chromatin was then assessed via PCR. Each analysis was performed in triplicate.

### Data analysis, protein sequence alignment and phylogenetic tree construction

All results shown represent an average of three replicates. Significant differences between groups were determined by Duncan’s new multiple range test, with significance tests denoted as i, ii, iii, and iv. Different lowercase letters above columns indicate significant differences (*P* < 0.05). The phylogenetic tree was constructed with MEGA 5.0, and related protein sequences were analyzed with Clustal X.

## Supplementary information

Supplemental Figure 1

Supplemental Figure 2
