# Supplementary figures and images for: MdMYB6 regulates anthocyanin formation in apple both through direct inhibition of the biosynthesis pathway and through substrate removal
Source: Hortic Res. 2020 May 2;7:72. doi: 10.1038/s41438-020-0294-4 (PMC7195469; doi:10.1038/s41438-020-0294-4)

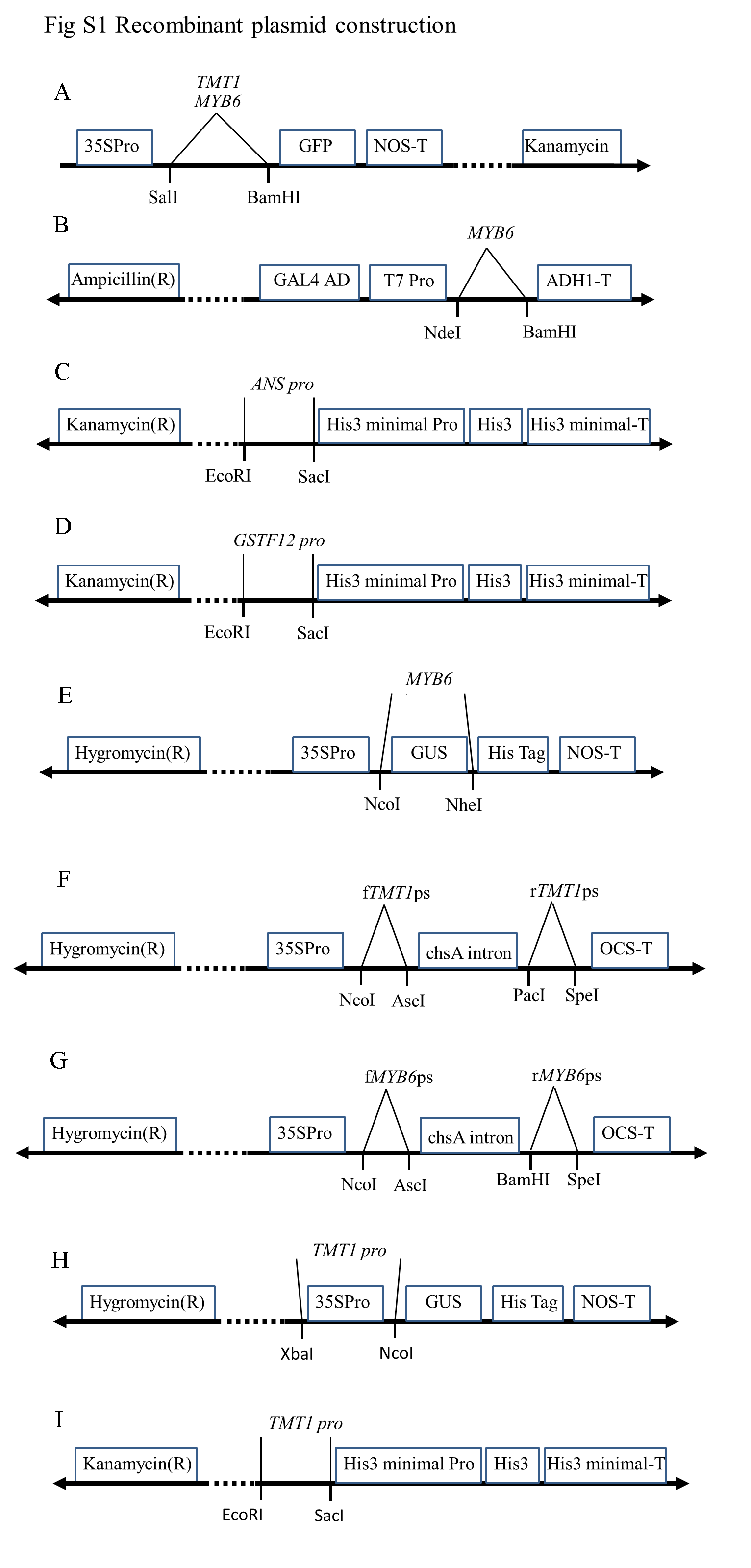

Supplement: Supplementary file 1 — Supplemental Figure 1 [file 41438_2020_294_MOESM1_ESM.tif]

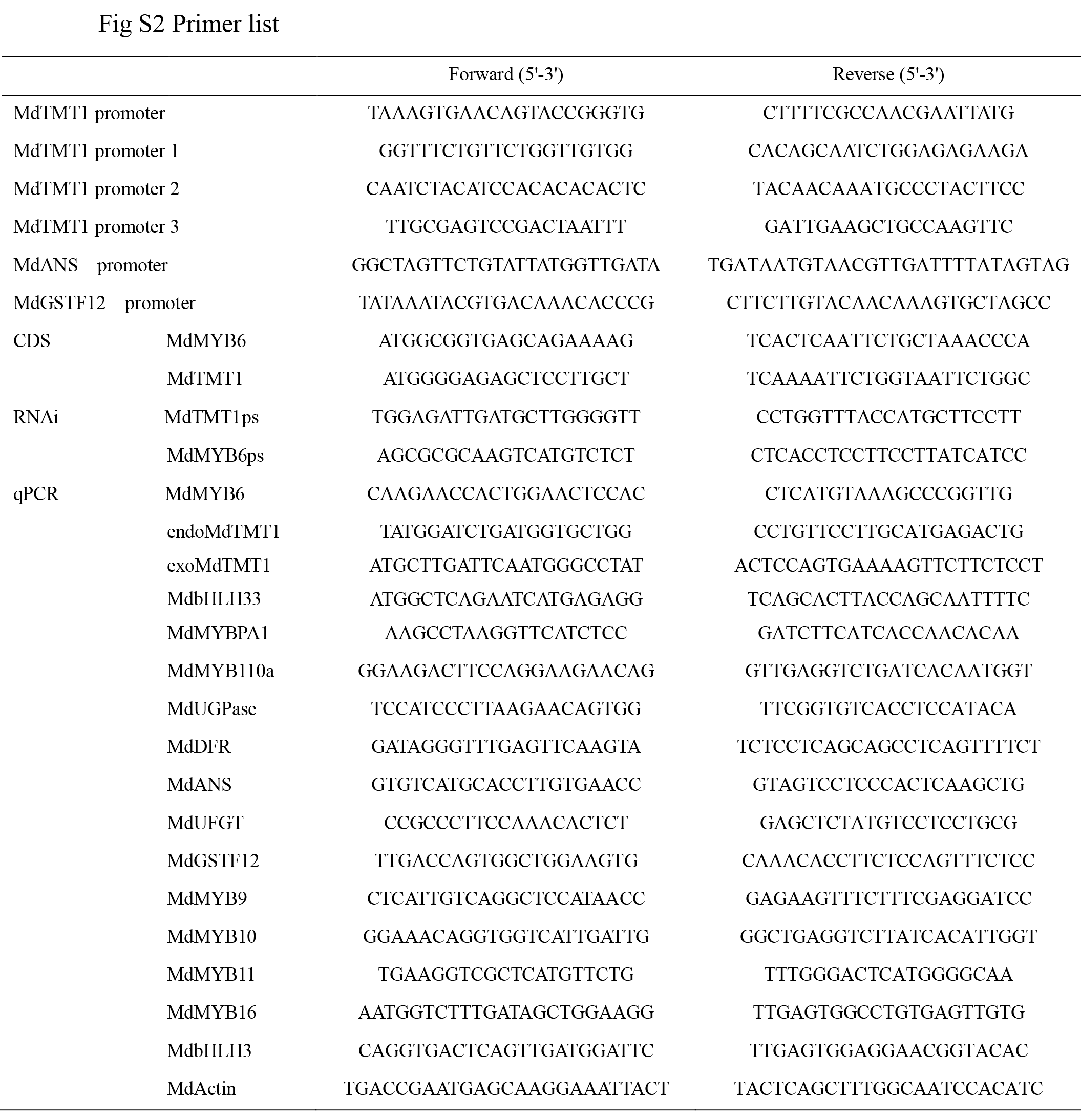

Supplement: Supplementary file 2 — Supplemental Figure 2 [file 41438_2020_294_MOESM2_ESM.tif]
